# Case of Puerperal Fever

**Published:** 1855-01

**Authors:** P. Van Patten

**Affiliations:** Walnut Camp, Arks.


### A CASE OF PUERPERAL FEVER.

BY P. VAN PATTEN, M. D.,

In approaching a case of Puerperal fever, I confess an inability to divest myself of an apprehension as to the termination of the attack, which the management and care of no other disease induces. All the phenomena and attendant circumstances are of a peculiarly exciting and painful interest. The history of the disease—the mournful tablet to which the anxious mind of the practitioner, reflexively and regretfully reverts, the moral associations, which, *ex necessitate*, are substantially identical in each individual instance, all combine to render it a matter of fearful import and highly unpleasant nature. No man possessing an ordinary modicum of the nobler sensibilities that dignify "poor human nature," still less, no legitimate and worthy disciple of Æsculapius and Hippocrates, can aught than desire to witness a reasonable and successful practice instituted in child-bed fever, an affection too justly classed among the *opprobria medicorum*. I say reasonable treatment, since it has escaped the observation of none, that in no other difficulty are there more temptations presented to pursue an experimental, (and toward the close) a vacillating course.

I wish not to obtrude *my* experience as a finality, nor *my* deductions as infallible; let them be criteria, only in so far as their correctness and utility are vindicated by collateral facts and extended observation; they are thrown out simply as passing notices to be accepted or rejected as my brethren deem most proper.

"A humble tribute to a worthy cause,
Is made less humble by its good intent."

As an exemplar of some of the graver features sometimes witnessed, I record the ensuing:

April 20th, called to see Mrs. M——, a lady of nervo-sanguine temperament, æt. 24. Two days previous to my visit, she was delivered of her second child, and being attended by an ignorant midwife, had suffered serious inconvenience from recklessness and mismanagement. The labor had been tedious, and from the particulars detailed me, it must have been a case of inertia of the uterus, depending upon an excessive accumulation of liquor amnii. Frictions, inunctions, *et id omne genus*, had been resorted to with an unsparing hand, with a vigor and pertinacity which would be commendable, were physical power and perseverance the essential elements of treatment demanded in emergencies of the nature considered. Under such violent and ill-advised manipulations the woman could not otherwise than suffer; the continued grasping of the abdominal parietes, the contusions experienced by the contiguous serous membranes and viscera, all tending to invite an undue afflux of blood, and establish the conditions provocative of inflammation. How sad to reflect that ignorance of rudimentary principles of treatment in these cases, involves a degree of suffering and danger of life which might all be obviated by a timely rupturing of the membranes, a simple and expeditious procedure.

I found her affected with acute pain in the hypogastric and iliac regions, with occasional lancinating pains in various parts of the abdomen; these upon pressure were aggravated to an intensity almost unendurable; skin hot to the feel, the pulse at 160 per minute, small and wiry—bowels constipated, with diminished secretion of urine. The lochia partially suppressed, considerable heat in the vagina and os uteri—heavy, dull pain in the lumbar region, tongue dry, and covered with a yellowish fur—thirst excessive. Decubitus, supine, with the knees slightly drawn up, countenance anxious and contracted. Here, indubitably, was a case of metro-peritonitis; I proceeded at once to venesection *pleno rivo*, until the pulse began to rise, feel softer, (it is important to recollect that a small, depressed and rapid pulse is not an evidence of extreme prostration in this disease,) and to become less frequent. About 40 oz. of blood were abstracted—a free Gordonian bleeding, it will be allowed. Under this treatment, the pulsations fell to 130, together with a cessation of delirium; the skin became somewhat moist, and within thirty minutes, there was less complaining of pain and general distress. Fomentations were next ordered—these to consist of woolen cloths dipped in boiling water, wrung dry, then to be folded two or three times to retain the heat, and applied over the whole extent of the abdomen ; to be frequently changed; likewise saline pediluvia, *pro re nata.* Several days having elapsed without any alvine dejections having been obtained, I deemed it best to waive the usual mercurial and opiate treatment, and give instead, after having quieted the stomach by the application of sinapisms over the scrobiculus cordis, etc.

℞ Proto-Chlor. Hydrarg. grs. xl.,
Jalap, Pulv. grs. x.,
Aqua, q. s.,
Misce,
S. To be taken at once.

Some advocate the opinion that a free catharsis is not to be induced in cases of peritoneal inflammation, on the ground that the peristaltic action of the intestines tend to aggravate the phlogosis, by inviting more blood and by disturbing the patient too much.—But surely any temporary afflux thus produced, would be amply compensated for, through the removal of a persistent cause of engorgement and inflammation, which a loaded and closely constipated bowel would inevitably involve—the consequent relief afforded to the absorbent system, &c., to say nothing of the sedative tendency in favor of the stomach and brain. In accordance with this view, after waiting three hours in vain, for some evidence of intestinal action, I exhibited three drops of Croton oil in a teaspoonful of syrup. At the expiration of an hour and a half, no catharsis being effected, the Croton oil was repeated and increased to five drops ; the response was prompt and free. The following was then exhibited.

℞ Proto-Chlor. Hydrarg, grs. xlv.,
Opii, Pulv., grs. xviij.,
Ft. Chart, jx.,
S. One to be taken every two hours.

The above to be continued until the narcotic effect be pretty strongly marked, and then to be given every three hours ; an effervescing draught to be used freely.

21st. I found my patient better. Skin moister, pulse at 120, respiration slower, pain still present but less severe, dorsal decubitus as before, nausea entirely gone, thirst slightly abated and the lochia returning. The powders had all been taken. This time I directed,

℞ Proto-Chlor. Hydrarg, grs. xl.,
Opii, Pulv., grs. xvj.,
Ipecac, grs. viij.,
Ft. Chart, viij.,
S. One to be taken every three hours.

Left with instructions if the pain, &c., became aggravated, to acquaint me with the fact.

22d. All indications decidedly favorable; no pain present ; some tenderness and flinching upon pressure, but this was to be expected. Skin moist, yellowish appearance of tongue changed to a slight whitish coating—pulse at 86, no nausea, diminished thirst, lochial discharge in sufficient quantity. The urine also was more copious and of lighter color, one alvine dejection had been obtained during the night. The anxious expression which previously characterized the countenance had yielded to a more placid appearance. Decubitus on the left side.

℞ Doveri, Pulv., grs. xviij.,
Quinine, Sulph., grs. xij.,
Magnesia, Calc., grs xx.,
Ft. Chart, vj.,
S. One to be taken every four hours.

This to be repeated the next day, late at night. Mass. Hydrarg. grs. xviij to be taken and followed with Ol Ricini, q. s., provided that no action be obtained by 8 A. M. of the following day. The effervescing draught with demulcent drinks to be used interchangeably. Thinking personal attendance no longer requisite, I directed Mr. M—— to acquaint me with progress day by day.

26th. I was sent for in haste to visit my patient again. She had been doing well in the interim since my last call until this morning ; her appetite was becoming good, febrile symptoms disappearing, and nothing unfavorable had manifested itself until a few hours before my arrival. Trusting too much to a rapid convalescence, she neglected my dietetic rules and indulged her appetite, and also imprudently exposed her health by rising from her bed and passing over a damp floor. Severe abdominal pains again present, emesis frequent and violent, the matter ejected being apparently bile and altered mucus of acrid character, pulse at 160 and very small, respiration at 36—lochia entirely suppressed, decubitus unfavorable. Here a question arose in my mind as to the propriety of further venesection. Although my patient was debilitated and the attack secondary, there were two considerations not to be lost sight of, viz: that the inflammation was confessedly sthenic, and my patient young and of full habit. The lancet was used, and some 20 oz. of blood abstracted, the pulse softer, attended with an increase of volume, and fell 40 pulsations within an hour, thus triumphantly vindicating the treatment. A blister was applied over the hypogastric and right iliac region, also sinapisms to the stomach, warm cloths to the vulva, pediluvia, &c., as before.—The subjoined was exhibited.

℞ Proto-Chlor. Hydrarg., grs. xxxij.,
Morph. Sulph., grs. lv.,
Ft. Chart, xij.,
S. One to be taken every two hours.

27th. My patient feels better. Gums tender, pulse ranging at 100, pain greatly diminished, very little nausea, skin not so torpid, bowels in a soluble condition, and the lochia returning, tongue not heavily coated, but rather too pointed and red at the tip. The ensuing was directed,

℞ Proto-Chlor. Hydrarg. grs. xxiv.,
Magnes. Calc., grs. jv.,
Opii Pulv., grs. xij.,
Ft. Chart, xij.,
S. One to be taken every two hours.

28th. Pulse at 94, soft and full. Pretty freely salivated, stomach quiet, with gentle diaphoresis, pain nearly absent, thirst moderate, bowels open—a general amelioration of all the symptoms. I prescribed accordingly. Upon visiting Mrs. M—— the next morning, she was as nearly as could be determined, in *statu quo*, though of course, relatively worse, (these stand points are not agreeable to the practitioner,) and here the case began to assume a more unfavorable aspect than at any time before. In a few hours the pulse rose to 120, notwithstanding the presence of what was deemed a judicious anti-phlogistic course, (active depletion being at this late stage inadmissible) and for several days occupied a varying range between that point and 100, accompanied with a partial return of the abdominal pain. Distressing and frequent vomiting was once more added to the list of symptoms. The tongue assumed a darker color, sordes accumulated on the teeth, respiration more frequent, with occasional singultus. Abdomen tympanitic, uterine discharges horribly fetid. The case was growing worse, the system cachectic; an asthenic inflammation had obtained. After a review of my therapeutics, I could not accuse myself of mal-practice, but the treatment *must* be modified. After applying "flying blisters" over the stomach, she was directed,

℞ Ol. Terebinth, ℥ss.,

Tr. Opii., gtt. xxx.,

Mucilage, ℥j.

Ft. Haust.

This to be repeated every four hours. Local applications, hygiene, &c., as at first. It would be tedious further to pursue the conduct of this case. By the use of opiates, terebinthinates, &c., with an *early exhibition of generous wine*, and light, but nutritious food, the lady recovered.

It may be thought that the treatment instituted was rather heroic. While the patient was before me I did not think so, nor can I at this date, upon a retrospect, believe it. Let us see. She bore depletion well, an incubus appeared to be thrown off the circulation —the biotic powers rallied; innervation, secretion and absorption conclusively proved it in each instance. Why a sthenic inflammation merged into an asthenic condition under such auspices, is more than I can divine; for evidently, the treatment was not wholly depletory and exhausting. Perhaps it is referable to a changed condition of the blood dependent upon the absorption of deleterious agents from the diseased tissues, added to depressing influences which a continuance of the disease would necessarily involve.—That the constitution of the atmosphere was a causative agent in this example, would be ruled out, on the ground of the indubitable fact that the inflammation was sthenic at its advent, and markedly so even after the relapse. Due attention had been given to the management of the sick room—no noxious effluvia had existence in the neighborhood—no local cause whatever, in so far as could be discovered, for the induction of adynamic manifestations. Due attention had been given to the removal of acrid substances from the vagina and uterus, by emollient injections, as I believe that negligence in this respect is apt to be attended with pernicious results.

It is true that an asthenic condition frequently follows its opposite; of this we have examples daily, but cases like that recorded above are rare. The sudden accession of graver symptoms, and the extreme prostration witnessed, could not have been anticipated reasonably, during the first and second stages. We must be satisfied with placing it among the inexplicable phenomena whose *arcana* may yet be patent; in the meantime deriving all the benefit from it which is practicable. Two practical hints may be deduced, viz: to watch the character of peritoneal inflammation jealously, and commence a roborant treatment early.

Walnut Camp, Arks., Sept. 6th, 1854.

